# Trends and Contributing Factors to Contraceptive Use in Kenya: A Large Population-Based Survey 1989 to 2014

**DOI:** 10.3390/ijerph17197065

**Published:** 2020-09-27

**Authors:** Asantesana Kamuyango, Wen-Hsuan Hou, Chung-Yi Li

**Affiliations:** 1 Department and Graduate Institute of Public Health, National Cheng Kung University, Tainan 701, Taiwan; asantesanak@gmail.com; 2 Department of Physical Medicine and Rehabilitation, Taipei Medical University Hospital, Taipei 110, Taiwan; houwh@tmu.edu.tw; 3 Master Program in Long-Term Care, College of Nursing, Taipei Medical University, Taipei 110, Taiwan; 4 Graduate Institute of Clinical Medicine, College of Medicine, Taipei Medical University, Taipei 110, Taiwan; 5 Center of Evidence-Based Medicine, Department of Education, Taipei Medical University Hospital, Taipei 110, Taiwan; 6 Department of Public Health, College of Public Health, China Medical University, Taichung 404, Taiwan; 7 Department of Healthcare Administration, College of Medical and Health Science, Asia University, Taichung 413, Taiwan

**Keywords:** contraception behavior, family planning services, facilities and services utilization, trends, social determinants of health, Kenya

## Abstract

Kenya is among the leading nations in family planning in Africa, having the first official nationwide family planning program in sub-Saharan Africa. However, Kenya is still one of the most highly populated countries in Africa with a population of more than 52 million. The objective of this study was to assess the trends and contributing factors of contraceptive use. We conducted a multi-wave cross-sectional study using both the demographic health survey (DHS) and family planning effort index (FPE) datasets, analyzing five-year waves from 1989 to 2014. This study indicates that contraceptive use increased from 24.0% to 42.6%, with a change % of 77.5%. Despite changes in women’s characteristics, these characteristics posed little on the time trend of contraceptive use in Kenya. In addition, the policy component of FPE scores had a positive association with contraceptive use with negligible change after adjusting for social and demographic factors 1.055 (1.046–1.065). There was a fluctuating trend of the additional FPE components throughout the years. Women with lower education, those married to husbands with lower education, unmarried, and rural women remain behind in family planning service utilization. Targeted programs are still needed for these special groups. Policy adherence is vital for continued progress.

## 1. Introduction

Contraceptive use benefits sexually active individuals to realize their fundamental right to choose freely and responsibly if, when, and how many children to have [1]. Most countries including developing countries have had national programs to provide family planning to large populations since the mid-1960s [2]. During the early years, advocates for family planning programs regularly highlighted their association with the economy, unlike today, where debates revolve around women’s reproductive health and rights [3]. Women experience a heavy burden on their physical and mental health due to unwanted pregnancies; therefore, there is a need to focus on their reproductive health [4]. Contraceptive use is essential to women’s general reproductive health, including post-rape care by a partner or non-partner [5]. Ever since the early 2000s, many United Nations member states have made considerable advancements towards improving contraceptive use and other reproductive health indicators [6]. Nevertheless, unintended pregnancy, resulting from the unmet need for contraception, still threatens the lives and wellbeing of women and their families globally, especially in low-income countries [7].

Kenya is regarded as one of the leading nations in family planning in Africa, having the first official nationwide family planning program in sub-Saharan Africa [8,9]. The Kenyan Government established the National Council for Population and Development (NCPD) in 1982, which has seen the fertility rate decline from 8.1 children per woman in 1977 to 6.7, 4.7, 4.6 and a remarkable 3.4 in 1989, 1998, 2008 and 2016, respectively [10,11]. Despite the change, family planning has constantly remained ‘business as usual’ as opposed to an emergency issue for policy elites [12]. Kenya is still one of the most highly populated countries in Africa, with a population of 52 million 574 thousand people in 2019 [13].

Previous studies had established that enhanced socioeconomic circumstances and robust family planning program efforts are vital in increasing contraceptive use [14]. Therefore, it is important to assess the trend and contributing factors of contraceptive use, as well as to estimate the role of family planning program efforts in determining contraceptive use. This secondary data analysis attempted to estimate the effects of other contributing factors on the trend of contraceptive use by using the characteristics of surveyed women from Kenya’s demographic health surveys (DHS) and the Family Planning Effort index (FPE).

## 2. Materials and methods

### 2.1. Data Sources

This study used both the DHS and FPE index datasets from Kenya. The FPE provides data by country downloaded from the Track20 website [15]. We obtained the permission from the Demographic Health Survey Program to use the survey datasets under the registered research project entitled “Contraceptive use in South-East Asia and Southern Africa”. The FPE is an internationally recognized scoring system for the four essential components of national family planning programs, namely: policy and stage-setting activities, service and service-related activities, record keeping and evaluation, and availability of contraceptive methods. The United Nations Population Division sponsors and recognizes this measure as an instrumental determinant of fertility within countries [16]. The study uses a rating scale of 1 to 10 (“1” for non-existent or very little effort and “10” for very strong effort) [2]. Program-specific detailed questionnaires are administered every five years to national experts on family planning [17]. The key informants include the ministry of health program staff, a native staff of nonprofit organizations, a resident staff of international organizations, and staff of local academic or research establishments. The interviews provide key insights from different perspectives on the progress of family planning-related interventions in the country [18]. We specifically used data from five-year rounds of data from 1989 to 2014, with the exception of 2004, when the FPI study was not recorded in Kenya.

The Demographic Health Surveys (DHS) system was established in 1984, as a tool for collecting nationally representative data on population, health, and nutrition from developing countries [19]. The National Statistical Offices (NSO) in developing countries implement the survey with funding from the United States Agency for International Development (USAID). The DHS offers cross-sectional survey data in five-year waves [19]. The DHS offers a nationally representative sample and it provides various indicators for social, economic, demographic, environmental, and health characteristics [20]. Kenyan demographic health surveys used a two stage sampling design with a response rate of 96% from 1989 to 2014 [21,22,23,24,25,26]. The DHS datasets are available to the general-public and downloaded upon request [27]. Highly trained interviewers collect the data in isolated standardized questionnaires from eligible adults. An individual dataset for women aged 15 to 49 years living in the sampled households was used in this study. The Demographic and Health Surveys (DHS) Program, 530 Gaither Road, Suite 500, Rockville, MD 20,850 USA approved our use for datasets from South-East Asia and Southern African countries.

### 2.2. Study Variables

We defined contraceptive use similarly as in previous publications based on DHS data. Contraceptive use was computed based on the responses given to the questions about the use of contraceptive methods. Women were asked the following questions (response options).

Current use by method type: (I) No method (II) Traditional method (III) Modern method. The outcome ‘current use by method type’ was transformed into a binary variable equal to “Yes” or “No”. Any method used was included in the definition of contraceptive use. We used eight variables for assessing social and demographic factors namely: age in years, marital status, age at first marriage, number of living children, woman’s education, husband’s education, Place of residence, and woman’s employment status. The variables from the FPE, namely total scores, policies, services, evaluation, and access, were added to the analysis by year, meaning that all study participants in a particular year had the same score as designed.

### 2.3. Statistical Analysis

The outcome variable and all independent variables were cross-tabulated by years. The percentage of change was calculated for each variable between 1989 and 2014. In addition, a test for trend was conducted using Cochran Armitage to assess the linear trend of various characteristics of study participants. Binary logistic regression models for the outcome (contraceptive use) were employed in two steps. We started by expressing the year variable as an independent categorical variable to assess the secular trend of contraceptive use (Model 1), then sequentially adjusting for the social and demographic factors in the subsequent models (Modes 2 and 3). To estimate the role of FPE, the next step included each component of the FPE separately to find the crude odds ratio of contraceptive use in association with each FPE component individually. We controlled for age, education, residence, employment status, and age at first marriage, marital status, and number of living children in the analysis of FPE effect on contraceptive use.

All results were weighted. We used a transformed individual weights variable formulated from v005/1,000,000 for frequencies. Complex samples package was used for regression analysis taking into consideration the primary sample unit, strata, and individual weights [28,29]. In 1989, strata variable was not given, therefore, strata were constructed by combining the type of residence variable and the household district variable according to survey design [21]. All analyses were conducted using IBM SPSS Statistics version 20 software.

### 2.4. Ethical Approval

This research has been approved by National Cheng Kung University Hospital. IRB approval code: B-ER-109-088.

## 3. Results

**Figure 1:**
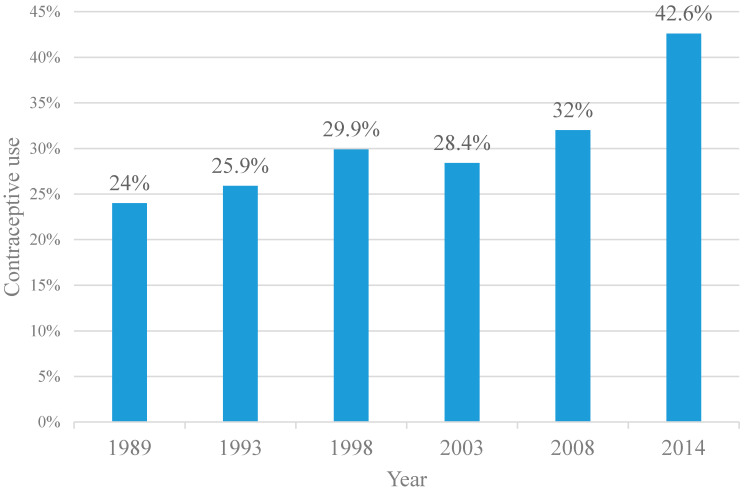
Contraceptive Use, Kenya, 1989–2014.

We analyzed 68,707 respondents with the number of respondents steadily increasing with each survey from 5568 in 1989 to 31,079 in 2014. During the study period, contraceptive use rates increased from 24.0% to 42.6%, with a change % of 77.5% (Figure 1).

**Figure 2:**
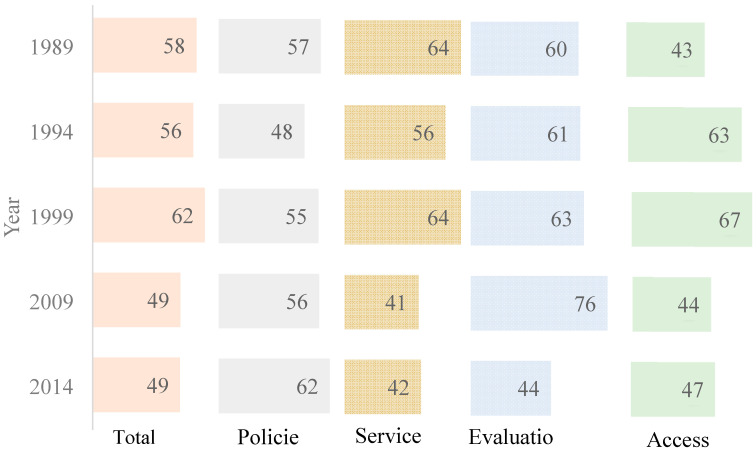
Trend in Family Planning Effort Index scores (FPE), 1989–2014.

The secular trends of contraceptive use were further stratified according to urbanization, women’s education, husband’s education, number of living children, working status, age at first marriage, marital status, and women’s age (Supplementary Figures S1–S8). With very few exceptions (e.g., women with higher education and husband with lower education), increasing contraceptive use was observed in all stratifications. The FPE index showed an increase in policy scores from 57 to 62. On the other hand, scores for services, evaluation, and access showed a fluctuating trend throughout the years (Figure 2).

The characteristics of women of reproductive age (15–49 years) are summarized by year in Table 1. The proportion of women with higher education increased from 0.3% to 11.2%, indicating a 3633% increase (*p* < 0.0001). In addition, urban residency increased by 135.8% (*p* < 0.0001). The percentage of women who did not work outside the home decreased from 88% to 38.5% (change %, −56.3%) (*p* < 0.0001). A notable decrease in women who were married before the age of eighteen was also observed, from 51.7% to 33.9% (change %, −34.4%) (*p* < 0.0001). The proportion of women with 9 or more children decreased from 2.7% to 0.5% (change %, −81.4%) (*p* < 0.0001).

Table 2 shows an increasing trend of contraceptive use by year. The crude odds ratio for contraceptive use was (1.104 (95% confidence interval (CI), 0.977–1.247) in 1993, (1.347 (95% CI, 1.203–1.509) in 1998, (1.252 (95% CI, 1.113–1.407) in 2003, (1.490 (95% CI, 1.329–1.672) in 2008 and (2.346 (95% CI, 2.136–2.577) using 1989 as the reference year. Entering the social and demographic factors in models 2 and 3 resulted in little change in the adjusted odds ratios by year of <2% when all variables are considered.

Marital status was shown to be the greatest determinant of contraceptive use, whereby married women had two times the odds of using contraceptives than unmarried women (adjusted odds ratio (AOR), 2.339 (95% CI, 2.104–2.600). In comparison to other ages, women aged above 25 had significant higher odds for contraceptive use, especially AOR, 1.591 (95% CI, 1.393–1.818) for 30–34-year-old, and (95% CI, 1.622; 95% CI, 1.428–1.841) for those aged 35–39 years. Those who married younger than eighteen years old were less likely to use contraceptives AOR (0.828 (0.775–884). Though statistically insignificant, this study also shows that those with four to six children, followed by those with between 7 to 9 living children, were more likely to use contraceptives (1.030 (0.795–1.333) and (1.005 (0.770–1.311), respectively. Living in urban areas (1.318 (1.222–1.422) and higher educational attainment (a woman’s and her husband’s) were associated with greater odds for contraceptive use, especially for women with university or higher degrees. However, those who were not currently working outside the home were less likely to use contraceptives (0.735 (0.689–0.785) (Table 3).

Table 3 shows the role of FPI scores, the policy component has a positive association with contraceptive use crude odd ratio (1.065 (1.058–1.073) and shows negligible change after adjusting for social and demographic factors 1.055 (1.046–1.065). On the other hand, the components of services, evaluation, and access showed a negative association with contraceptive use after adjusting for social and demographic factors (0.973 (0.969–0.977), (0.978 (0.974–981) and (0.947 (0.941–0.952). A similar result could be seen for the total program index (0.954 (0.948–0.962).

## 4. Discussion

Our analysis showed steady growth in contraceptive use from 24% to 42.6% from 1989–2014 in Kenya. These results provide the opportunity for a long-term analysis of contraceptive time trends in a developing country, based on the indicator, which is strictly aligned with the sustainable development goals (SDGs) to guarantee universal access to family planning and education [30]. It is important to note that the slight decline in the trajectory in 2003 could have been as a result of a delayed response to the transitions in funding from fully donor funded programs into need for Kenyan government’s contribution [12]. The time trend in the increase in contraceptive use is possibly associated with Health sector reform policies and strategies adopted by the Kenyan government. These include the Kenya Health Policy Frameworks [31,32] and strategic plans known as the National Health Sector Strategic Plans (NHSSP) [33,34,35] renewed every five years since 1994. Though some would argue that individual interventions play a part in this reform, the improvements in health financing and service delivery form a large extent of the population-level improvements [36]. In the year 2000, Kenya adopted its new Population Policy which prompted the government to make renewed efforts towards family planning after declining donor resource allocation [12]. In addition, Kenya piloted a voucher program to subsidize family planning services for poor women since 2006 [37]. Performance-based incentive (PBI) programs were introduced to enhance intrinsic motivation by rewarding Family Planning providers for providing quality services [38]. The National Reproductive Health Strategy covering 2009 to 2015 recognized Community Health Workers as the first level providers for family planning services with increased donor funding during the same period [33,35]. All these initiatives increased access to and acceptance of family planning services over time.

We also observed noteworthy time trends in women’s characteristics associated with contraceptive use [39]. In the course of 25 years from 1989–2014, we observed an increase in women with a university degree or higher education, those whose husbands had a higher degree and those employed outside the home. We also observed a reduction in those who lived in rural areas, those who were married below the age of 18, and those who had more than 9 children. Such a major shift in women’s social standing has been consistently observed over recent decades in sub-Saharan Africa [40]. Despite dramatic changes in women’s characteristics, these characteristics had little impact on the time trend of contraceptive use in Kenya. The significant difference in contraceptive use between married and unmarried women corresponds with other studies [41], whereby married women have been observed to have the need for family planning than single women. A woman’s and her husband’s secondary and tertiary level of education presented an increased likelihood for contraceptive use, which is consistent with other studies [42], since higher education offers women an enhanced perspective on contraceptive choices including accessibility [43]. Educated women being more likely to be employed also meant that those who were currently working outside the home were more likely to use contraceptives compared to those who did not. Our study corresponds with other studies in southern Africa that show the non-use of contraceptives among younger women [44]. Besides the recent increase in contraceptive use among those between the ages of 20–24, cultural stigma and lack of youth-friendly health services are the biggest challenges for contraceptive use among younger women [45]. Urban and rural disparities in contraceptive use have been persistent through the years, as has been established in previous studies [46]. Lack of access to and knowledge concerning reproductive health put rural women at a disadvantage compared to their urban counterparts [47].

Program effort under each category fluctuated throughout the years, as well as showing improvement compared to other countries within sub-Saharan Africa [48]. However, only the policy component was shown to be positively associated with contraceptive use among women. This could be as a result of the policy component being the one that showed the least fluctuation and a positive percentage increase between 1989–2014. These findings support other studies that showed that strong policies are very essential in increasing contraceptive use [49]. In Kenya, policy documents are well written and supported by accurate data; however, the implementation of policies has been a challenge [50]. The gap in implementation could also result from lack of resources, a weak logistic and delivery system, and local level differences in implementation since the decentralization process in 2013 [12,51]. The other FPE components are very dependent on the robust implementation of set policies thereby could be largely affected by political unrest and economic fluctuations, for instance, the 2008 post-election violence [45]. Funding is also a persistent challenge in Kenya, making it difficult to sustain interventions over time [51]. Services, evaluation, and access reveal a negative association with contraceptive use, suggesting the complex nature of the interrelationship between program efforts and contraceptive use. There is an essential need to keep in mind the evolving nature of political, cultural and social circumstances in women’s lives, making it difficult for us to achieve a general trend in the relationship between society-level interventions and individual women’s contraceptive use [52].

The most prominent strength of this study is the large sample size covering several decades’ worth of multi-wave cross-sectional studies. To our knowledge, there has not been a country-specific study using both the DHS and FPE data to examine contraceptive use over such an extensive timeline. This study has offered us an opportunity to examine the trend of contraceptive use in Kenya since 1989.

We also acknowledge several limitations in our study. Firstly, the cross-sectional nature of both datasets means that we cannot make causal inferences for these findings. The years of demographic health data collection did not exactly align with the family planning program effort index, which meant that there was sometimes a year difference between the two corresponding survey results. Secondly, the FPE index scores were not collected for the year 2004, which meant that there were no corresponding program effort results for women surveyed in the Demographic health survey of 2003. Third, this study has not taken into account community-level variables, which would further explore multilevel dimensions that influence contraceptive use among women. Fourth, the DHS is based on self-report data, which are vulnerable to recall bias and social desirability bias. Last, the information on the FPE index is entirely based on the responses of the key informants. As such, the information might be biased by an informant’s knowledge of key outcomes (contraceptive use and fertility decline). If contraceptive prevalence is high, the respondents might unconsciously give high scores to the availability of methods. Despite these limitations, these analyses provide valuable insights on the trends and contributing factors of contraceptive use in Kenya.

## 5. Conclusions

This article updates the current knowledge on the trend of contraceptive use in Kenya. Bridging the gap between policy and implementation is needed to see a sustained increase in contraceptive use in Kenya. Women with lower education, those married to husbands with lower education, unmarried, and rural women remain behind in family planning service utilization. Targeted programs are needed to see improvements in these special groups. Future research must focus on specific program efforts and their role in specified dimensions of contraceptive use.

## Figures and Tables

**Table 1:** Characteristics of women in reproductive age—Kenya Demographic Health Survey 1989–2014 (five year waves) (total *n* = 68,707) ^a^.

	Calendar Year							
	1989	1993	1998	2003	2008	2014		
Characteristics	*n* = 5568	*n* = 7540	*n* = 7881	*n* = 8195	*n* = 8444	*n* = 31,079	Change (%)	*p*-Value ^b^
	%	%	%	%	%	%		
Age in years								
15–19	20.5%	23.3%	23.5%	22.6%	20.9%	18.7%	−8.7	3.128
20–24	18.0%	21.7%	19.6%	20.6%	20.3%	18.5%	2.7	0.001↑
25–29	19.2%	16.2%	17.4%	16.9%	17.2%	19.6%	2.0	7.783
30–34	14.3%	14.4%	12.5%	13.3%	14.3%	14.5%	1.3	0.042↑
35–39	12.5%	10.2%	12.6%	10.6%	10.4%	12.1%	−3.2	0.183
40–44	9.2%	8.5%	8.1%	9.6%	9.1%	9.3%	1.0	0.030↑
45–49	6.3%	5.8%	6.3%	6.4%	7.8%	7.3%	0.1	2.813
Currently married	67.0%	61.4%	61.3%	60.0%	58.4%	59.7%	−10.8	7.317
Marriage age <18 yrs.	51.7%	45.9%	42.6%	38.9%	37.8%	33.9%	−34.4	0.000↓
Number of living children								
None	23.2%	29.0%	28.9%	29.3%	28.4%	26.6%	14.6	0.725
1–3	34.0%	35.5%	39.7%	41.5%	43.2%	47.9%	40.8	0.000↑
4–6	27.0%	23.0%	22.2%	21.6%	21.8%	20.2%	−25.1	2.641
7–9	13.2%	10.6%	8.2%	6.8%	5.7%	4.7%	−64.3	0.000↓
>9	2.7%	1.8%	1.0%	0.8%	0.9%	0.5%	−81.4	6.185
Woman’s education								
No education	25.5%	17.9%	11.5%	12.7%	8.9%	7.0%	−72.5	0.000↓
Primary	54.6%	57.6%	59.2%	58.0%	56.8%	50.3%	−7.8	1.473
Secondary	19.5%	23.9%	26.9%	23.5%	26.9%	31.5%	61.5	0.000↑
Higher	0.3%	0.6%	2.3%	5.9%	7.3%	11.2%	3633	0.000↑
Husband’s education								
No education	16.6%	12.9%	9.1%	11.6%	8.7%	6.8%	−59.0	0.000↓
Primary	51.5%	52.0%	48.6%	48.4%	47.9%	47.0%	−8.7	2.76
Secondary	30.4%	33.5%	37.4%	30.3%	33.0%	32.8%	7.8	0.665
Higher	1.5%	1.6%	4.9%	9.8%	10.3%	13.4%	793.3	0.000↑
Residence (urban)	17.3%	17.8%	23.2%	25.1%	25.4%	40.8%	135.8	0.000↑
Currently (not)working	88.0%	51.0%	48.1%	41.5%	43.2%	38.5%	−56.3	0.000↓

^a^ This analysis uses the Kenya DHS 1989–2014, including survey weights, and is therefore representative of the national population. ^b^ Based on Cochran–Armitage test).

**Table 2:** Odds ratios and 95% confidence interval of contraceptive use in relation to calendar year as well as social and demographic factors in Kenya from 1989 to 2014.

Year and Women’s Characteristics	Contraceptive Use (%)	Model 1 ^a^	Model 2 ^a^	Model 3 ^a^
	*n* = 23,911	COR (95% CI)	AOR (95% CI)	AOR (95% CI)
Year				
1989	1338 (24.0)	Reference	Reference	Reference
1993	1951 (25.9)	1.104 (0.977–1.247)	1.263 (1.090–1.464)	1.029 (0.882–1.199)
1998	2355 (29.9)	1.347 (1.203–1.509)	1.582 (1.380–1.815)	1.146 (0.994–1.323)
2003	2324 (28.4)	1.252 (1.113–1.407)	1.563 (1.352–1.807)	1.142 (0.988–1.321)
2008	2705 (32.0)	1.490 (1.329–1.672)	1.957 (1.698–2.254)	1.362 (1.183–1.569)
2014	13,238 (42.6)	2.346 (2.136–2.577)	3.169 (2.826–3.552)	2.070 (1.824–2.349)
Age group in years				
15–19	1144 (8.1)		1.024 (.858–1.220)	0.901 (0.731–1.111)
20–24	4247 (31.9)		1.237 (1.100–1.390)	1.057 (0.915–1.221)
25–29	5688 (45.2)		1.580 (1.421–1.757)	1.351 (1.183–1.542)
30–34	4753 (49.1)		1.814 (1.633–2.015)	1.591 (1.393–1.818)
35–39	3841 (48.2)		1.797 (1.623–1.990)	1.622 (1.428–1.841)
40–44	2678 (43.0)		1.575 (1.414–1.755)	1.537 (1.343–1.759)
45–49	1559 (33.0)		Reference	Reference
Currently married	19,360 (46.6)		2.408 (2.219–2.613)	2.339 (2.104–2.600)
Marriage age < 18 yrs.	7002 (37.2)		0.686 (0.650–0.724)	0.828 (0.775–0.884)
Number of living children				
None	1514 (8.0)		0.204 (0.152–0.274)	0.122 (0.087–0.170)
1–3	13,605 (45.9)		1.431 (1.123–1.823)	0.852 (0.651–1.115)
4–6	6925 (46.5)		1.415 (1.120–1.787)	1.030 (0.795–1.333)
7–9	1670 (35.7)		1.091 (0.863–1.379)	1.005 (0.770–1.311)
>9	196 (29.9)		Reference	Reference
Woman’s education				
No education	1281 (16.8)			0.305 (0.246–0.378)
Primary	13,012 (34.9)			0.672 (0.558–0.808)
Secondary	7361 (38.7)			0.964 (0.800–1.162)
Higher	2254 (46.8)			Reference
Husband’s Education				
No education	582 (15.9)			0.375 (0.311–0.453)
Primary	6855 (38.8)			0.756 (0.656–0.871)
Secondary	5719 (48.0)			0.889 (0.774–1.021)
Higher	1751 (59.7)			Reference
Residence (urban)	8607 (40.9			1.318 (1.222–1.422)
Currently (not) working	5661 (22.5)			0.735 (0.689–0.785)

^a^ Complex samples package was used for regression analysis taking into consideration the primary sample unit, strata, and individual weights.

**Table 3:** Odds ratios and 95% confidence interval of contraceptive use in relation to program index scores in Kenya from 1989 to 2014.

Program Index								Total	
Policies		Services		Evaluation		Access			
COR (95% CI)	AOR ^a^ (95% CI)	COR (95% CI)	AOR ^a^ (95% CI)	COR (95% CI)	AOR ^a^ (95% CI)	COR (95% CI)	AOR ^a^ (95% CI)	COR (95% CI)	AOR ^a^ (95% CI)
1.065 (1.058–1.073)	1.055 (1.046–1.065)	0.969 (0.966–0.972)	0.973 (0.969–0.977)	0.973 (0.970–0.976)	0.978 (0.974–0.981)	0.979 (0.976–0.982)	0.982 (0.978–0.986)	0.947 (0.941–0.952)	0.954 (0.948–0.962)

^a^ Adjusted for Age, education, residence, employment status, age at first marriage, marital status, and number of living children.

## Data Availability

The original datasets used in this study are available upon request from the Demographic Health Surveys and track20 website respectively.

## Supplementary Materials

The following are available online at https://www.mdpi.com/1660-4601/17/19/7065/s1, Figure S1: Contraceptive use by type of residence, Figure S2: Contraceptive use by women’s education, Figure S3: Contraceptive use by husband’s education, Figure S4: Contraceptive use by number of living children, Figure S5: Contraceptive use by working status, Figure S6: Contraceptive use by age at first marriage, Figure S7: Contraceptive use by marital status, Figure S8: Contraceptive use by age group.
