# Supplementary material for: Trends and Contributing Factors to Contraceptive Use in Kenya: A Large Population-Based Survey 1989 to 2014
Source: Int J Environ Res Public Health. 2020 Sep 27;17(19):7065. doi: 10.3390/ijerph17197065 (PMC7579622; doi:10.3390/ijerph17197065)
Supplement: Supplementary file 1 [file ijerph-17-07065-s001.pdf]

International Journal of
Environmental Research and Public Health

Supplementary Materials

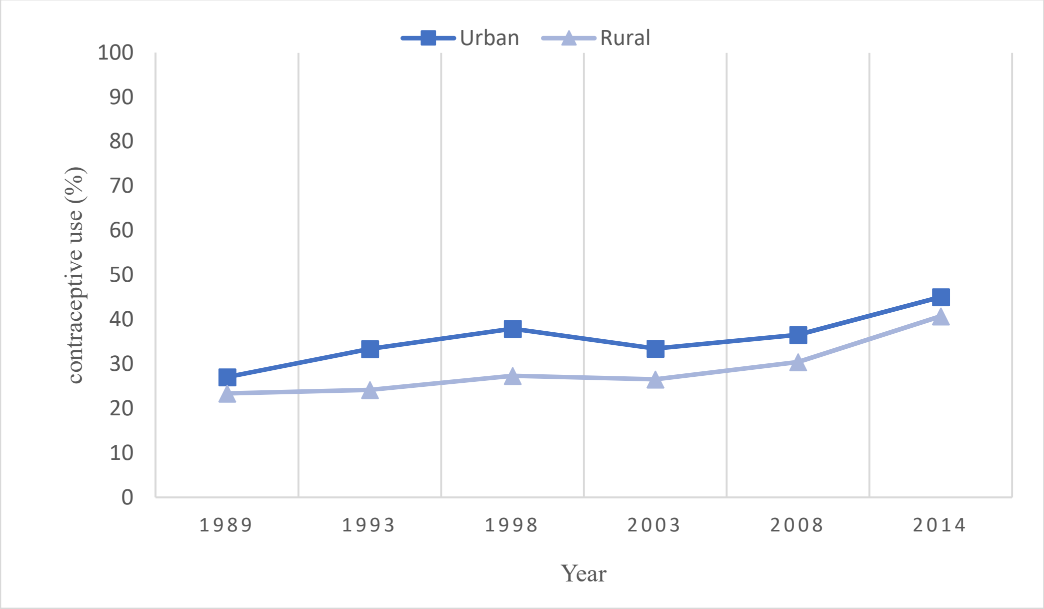

Supplement Figure 1. Contraceptive use by type of residence.

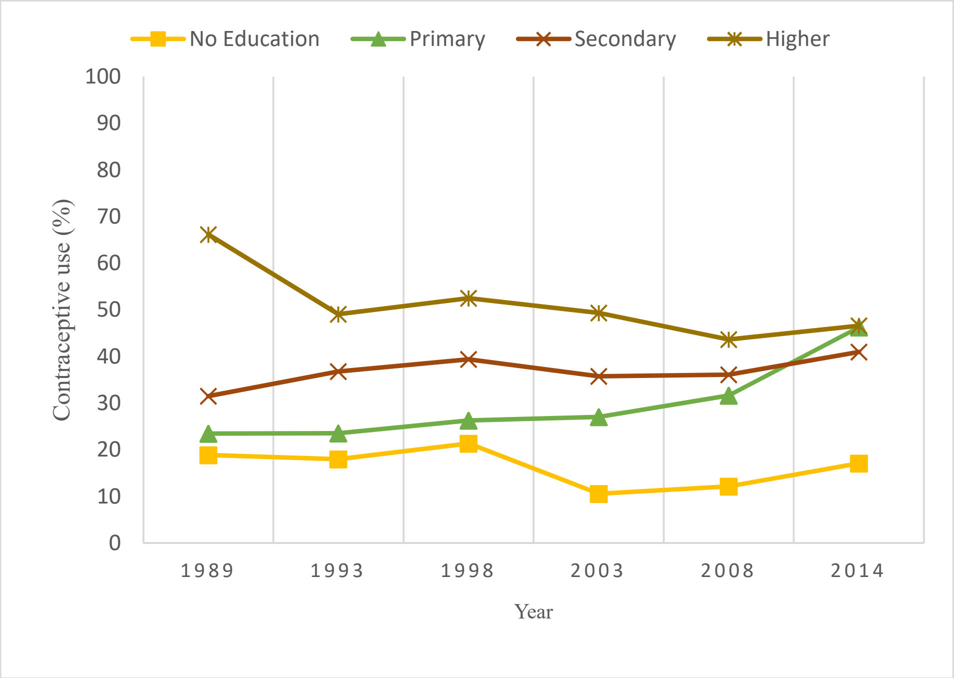

Supplement Figure 2. Contraceptive use by women's education.

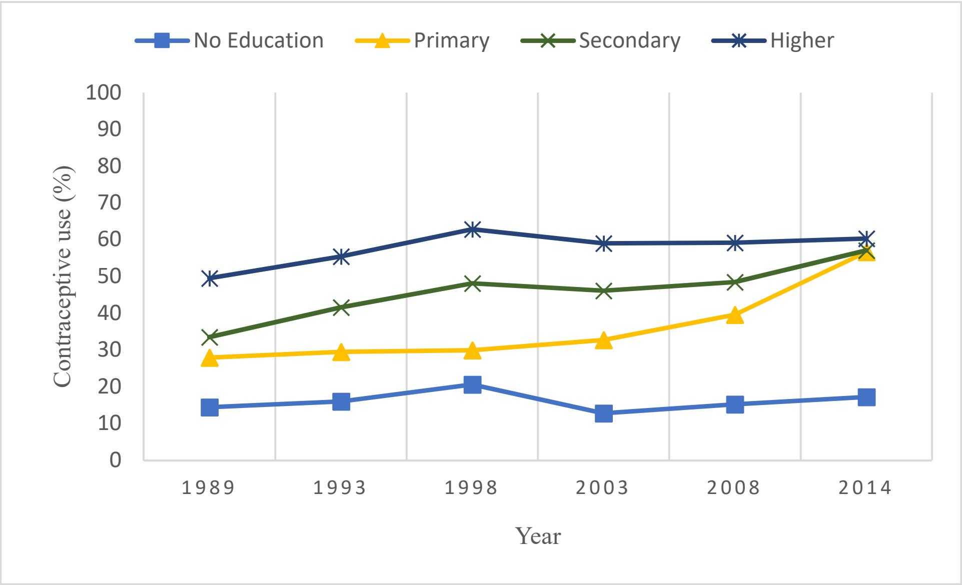

Supplement Figure 3. Contraceptive use by husband's education.

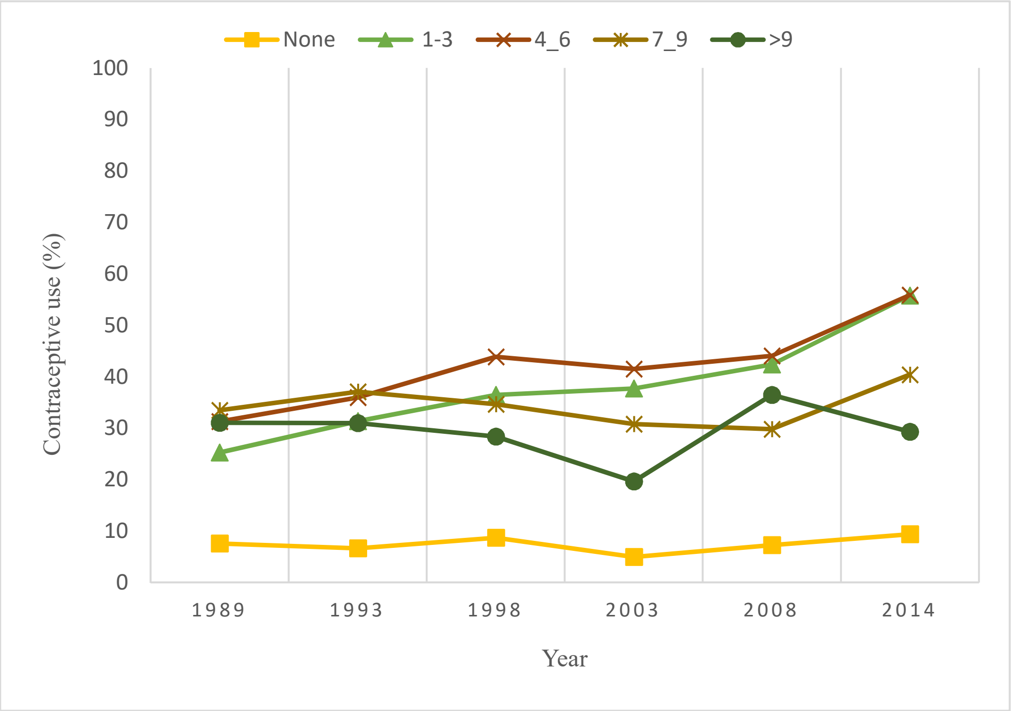

Supplement Figure 4. Contraceptive use by number of living children.

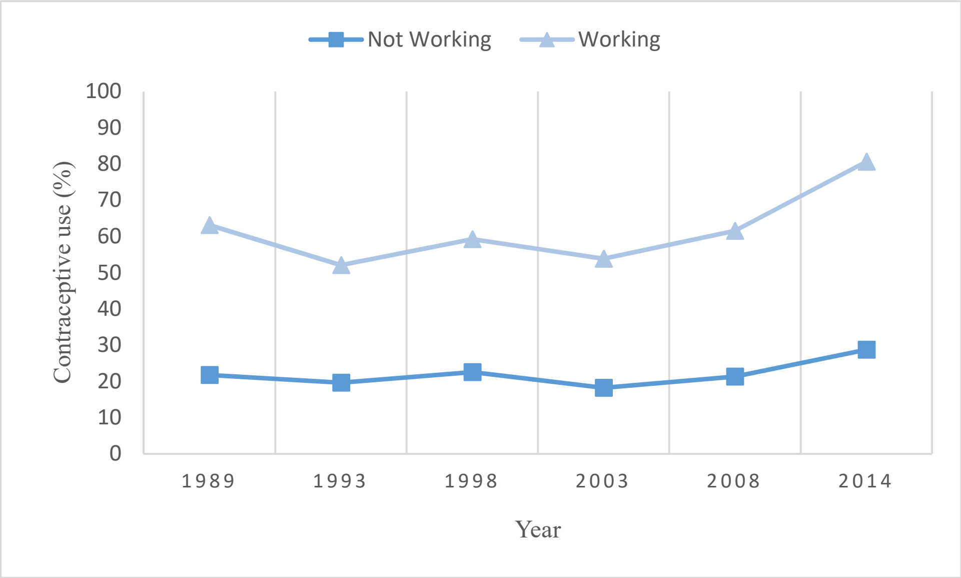

Supplement Figure 5. Contraceptive use by working status.

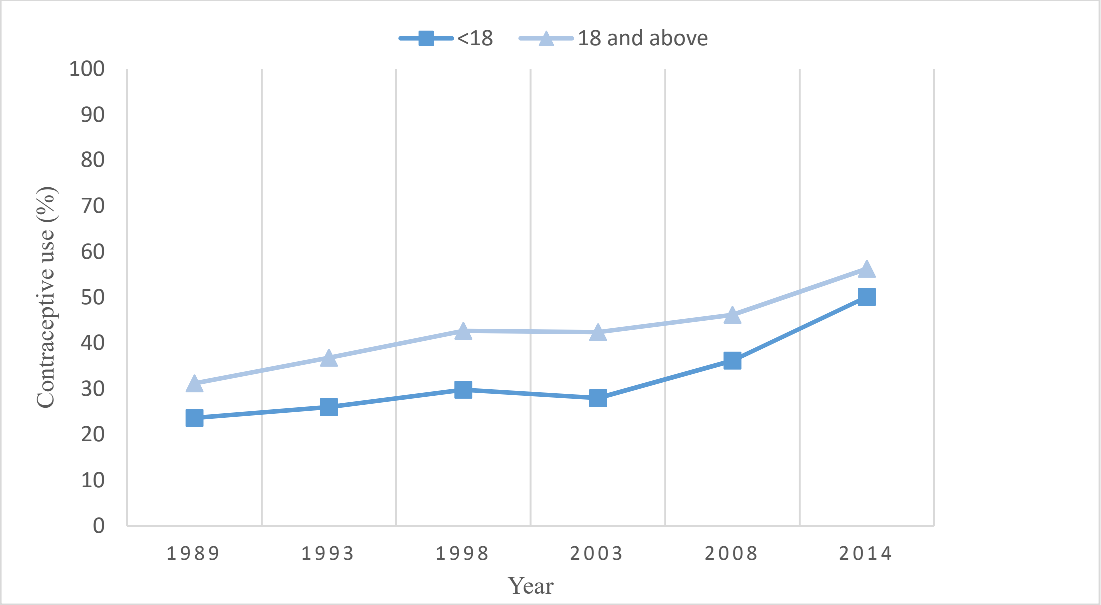

Supplement figure 6. Contraceptive use by age at first marriage.

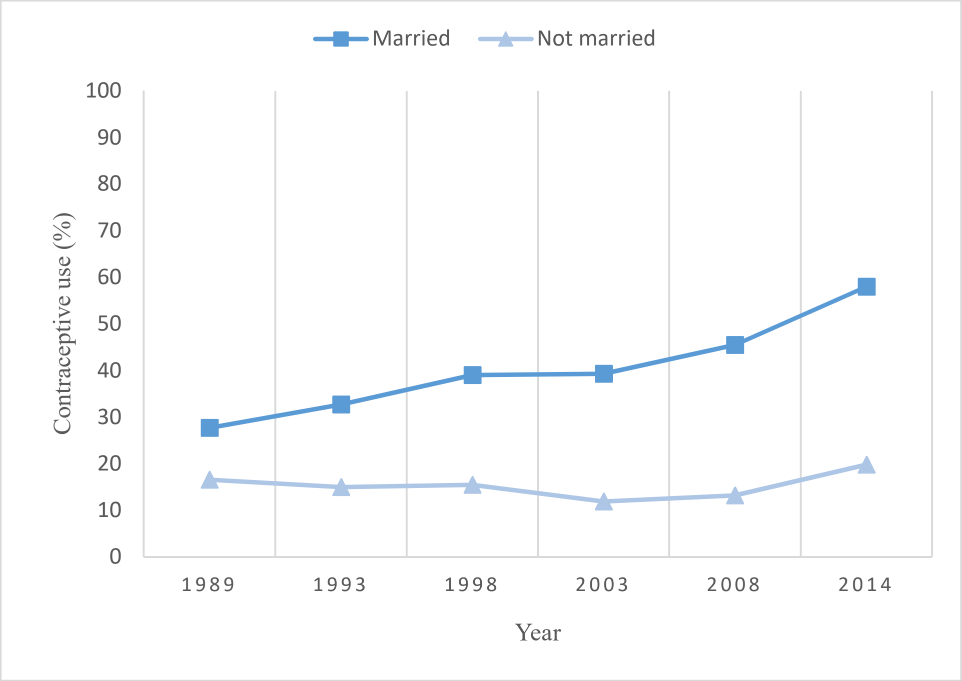

Supplement Figure 7. Contraceptive use by marital status.

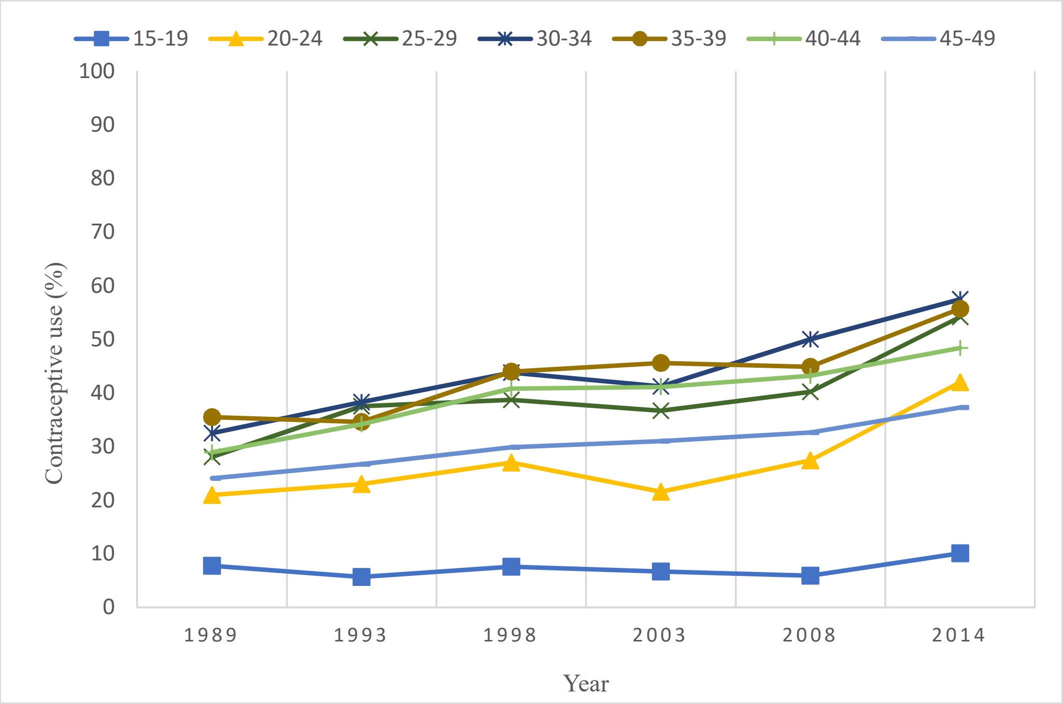

Supplement Figure 8. Contraceptive use by age group.
